# Prehospital Care for the Adult and Pediatric Seizure Patient: Current Evidence-based Recommendations

**DOI:** 10.5811/westjem.2016.12.32066

**Published:** 2017-03-03

**Authors:** Eric C. Silverman, Karl A. Sporer, Justin M. Lemieux, John F. Brown, Kristi L. Koenig, Marianne Gausche-Hill, Eric M. Rudnick, Angelo A. Salvucci, Greg H. Gilbert

**Affiliations:** * University of California, San Francisco, School of Medicine, Department of Emergency Medicine, San Francisco, California; † University of California, San Francisco, Department of Emergency Medicine, San Francisco, California; ‡ Stanford School of Medicine, Department of Emergency Medicine, Stanford, California; § University of California, Irvine, School of Medicine, Department of Emergency Medicine, Irvine, California; ¶ University of California, Los Angeles, David Geffen School of Medicine, Department of Emergency Medicine, Los Angeles, California; || Harbor-UCLA Medical Center, Department of Emergency Medicine, Torrance, California; # EMS Medical Directors Association of California

## Abstract

**Introduction:**

We sought to develop evidence-based recommendations for the prehospital evaluation and treatment of adult and pediatric patients with a seizure and to compare these recommendations against the current protocol used by the 33 emergency medical services (EMS) agencies in California.

**Methods:**

We performed a review of the evidence in the prehospital treatment of patients with a seizure, and then compared the seizure protocols of each of the 33 EMS agencies for consistency with these recommendations. We analyzed the type and route of medication administered, number of additional rescue doses permitted, and requirements for glucose testing prior to medication. The treatment for eclampsia and seizures in pediatric patients were analyzed separately.

**Results:**

Protocols across EMS Agencies in California varied widely. We identified multiple drugs, dosages, routes of administration, re-dosing instructions, and requirement for blood glucose testing prior to medication delivery. Blood glucose testing prior to benzodiazepine administration is required by 61% (20/33) of agencies for adult patients and 76% (25/33) for pediatric patients. All agencies have protocols for giving intramuscular benzodiazepines and 76% (25/33) have protocols for intranasal benzodiazepines. Intramuscular midazolam dosages ranged from 2 to 10 mg per single adult dose, 2 to 8 mg per single pediatric dose, and 0.1 to 0.2 mg/kg as a weight-based dose. Intranasal midazolam dosages ranged from 2 to 10 mg per single adult or pediatric dose, and 0.1 to 0.2 mg/kg as a weight-based dose. Intravenous/intrasosseous midazolam dosages ranged from 1 to 6 mg per single adult dose, 1 to 5 mg per single pediatric dose, and 0.05 to 0.1 mg/kg as a weight-based dose. Eclampsia is specifically addressed by 85% (28/33) of agencies. Forty-two percent (14/33) have a protocol for administering magnesium sulfate, with intravenous dosages ranging from 2 to 6 mg, and 58% (19/33) allow benzodiazepines to be administered.

**Conclusion:**

Protocols for a patient with a seizure, including eclampsia and febrile seizures, vary widely across California. These recommendations for the prehospital diagnosis and treatment of seizures may be useful for EMS medical directors tasked with creating and revising these protocols.

## INTRODUCTION

Seizures are a common medical condition, with 10% of Americans experiencing at least one seizure in their lifetimes and epilepsy developing in 3% by the age of 75. In the United States (U.S.), approximately 200,000 new cases of epilepsy are diagnosed each year, with the highest incidence among individuals younger than two years and older than 65 years of age.^1^ Seizure evaluation and treatment makes up a significant portion of emergency medical services (EMS) utilization, accounting for 5 – 8% of all EMS calls.^2^ Approximately 71% of these calls result in EMS transport and make up approximately 1.2% of all emergency department (ED) visits.^3^ Prehospital interventions, such as airway management, establishing intravenous (IV) access, benzodiazepine administration and blood glucose testing are commonly performed.^4^ In one study of 140 EMS providers across 40 states, prehospital treatment with a benzodiazepine was observed in 8.3% of seizure cases.^4^ While advanced life support (ALS) care is common in prehospital seizure management, there are a broad range of interventions employed.

### Status Epilepticus

Status epilepticus (SE) is defined as prolonged seizures (greater than five minutes), or multiple seizures without return to baseline between episodes. SE is a relatively common seizure disorder, with an annual incidence of approximately 40 per 100,000.^5^ The occurrence of SE has tripled in the past 30 years, with approximately 60,000 new cases annually in the U.S.^5^,^6^ SE has a significant impact on individual patients as well as the entire healthcare system. Although there have been substantial improvements in the treatment of SE, the overall mortality remains at approximately 20%.^5^–^7^ The annual inpatient financial costs of SE are estimated at more than $4 billion in the U.S. alone.^5^,^8^

Although SE remains a significant source of morbidity and mortality, research has shown that appropriate prehospital treatment improves patient outcomes.^9^ The optimal prehospital management of seizures continues to evolve as new medications and routes of administration are introduced. Without widely accepted guidelines, however, EMS care continues to vary greatly across the U.S. The Institute of Medicine report, “Emergency Medical Services at the Crossroads,” notes that EMS needs more uniform, high quality care and specific standards for evaluating that care.^10^ One such standard is the prehospital protocol that EMS providers use while caring for patients in the field. As such, we aim to provide a summary of the evidence for the prehospital evaluation and treatment of patients with seizure, and to assess the consistency of California protocols.

## METHODS

The State of California divides EMS care into 33 local EMS agencies (LEMSAs), which are geographically divided governmental regulatory bodies. One set of governmental medical control policies regulates the first responders and ambulance transporters in each countywide or region-wide system, in accordance with EMS Authority scope of practice. Medical directors of those agencies, along with other EMS medical directors, make up the EMS Medical Directors Association of California (EMDAC). EMDAC supports and guides the various agencies and makes recommendations to the California EMS Authority about policy, legislation and scope of practice issues. In an effort to improve quality and decrease variability in EMS practice in California, EMDAC has endeavored to create evidence-based recommendations for EMS protocols. Those recommendations and previous reviews are intended to assist medical directors of the various local EMS agencies to develop high quality, evidence-based protocols.

**Population Health Research Capsule**

What do we already know about this issue?

Seizure evaluation and treatment makes up a significant portion of EMS utilization; however, high-quality, specific guidelines have not yet been widely accepted.

What was the research question?

How do the prehospital seizure protocols of each of the 33 EMS agencies in California compare?

What was the major finding of the study?

EMS protocols for a patient with a seizure, including eclampsia and febrile seizures, vary widely across CA.

How does this improve population health?

Recommendations for the prehospital diagnosis and treatment of seizures may be useful for EMS medical directors tasked with creating and revising these protocols.

A subcommittee of EMDAC developed this manuscript and chose by consensus the elements that should be included in any protocol for a patient with a suspected seizure. The subcommittee then created a narrative review of the existing evidence for prehospital treatment of seizures. Clinical questions regarding those interventions were developed in the PICO (population, intervention, control and outcome) format. Our population included those patients in the prehospital setting with a suspected seizure. The intervention varied by clinical question. The control consisted of patients who were not receiving the specific intervention, and outcomes were defined by cessation of seizure activity after intervention.

We relied on recommendations made by various organizations that have performed systematic reviews and meta-analyses regarding treatment interventions, including the Neurocritical Care Society and the Cochrane Collaboration. We supplemented these recommendations with additional literature searches through PubMed from 1966 to 2016 for each question. During our primary literature review of PubMed, we searched for the terms “prehospital and seizure,” “status epilepticus,” “eclampsia,” and “febrile seizure.” That search yielded 161 articles, 59 of which were published in English and pertinent to the topics identified by the EMDAC subcommittee. It was supplemented with additional PubMed searches for specific topics.

We assigned levels of evidence (LOE) and graded our recommendations based on the American College of Emergency Physicians (ACEP) process of creating their clinical policies with slight modification to better fit our objectives.^11^ This committee of EMDAC reviewed studies and assigned LOEs based on the study design, including features such as data collection methods, randomization, blinding, outcome measures and generalizability. LOE I consisted of randomized, controlled trials, prospective cohort studies, meta-analysis of randomized trials or prospective studies or clinical guidelines/comprehensive review. LOE II consisted of nonrandomized trials and retrospective studies. LOE III consisted of case series, case reports, and expert consensus. After assigning LOEs to the studies, we translated those to clinical grades of recommendations using the following standards:

Level A Recommendations: Prehospital recommendations with a strong degree of certainty based on one or more LOE I studies or multiple LOE II studies.
Level B Recommendations: Prehospital recommendations with a moderate degree of certainty based on one or more LOE II studies or multiple LOE III studies.
Level C Recommendations: Prehospital recommendations based on only poor quality or minimal LOE III studies or based on consensus.

No Recommendation: No recommendation was given in those cases where only preliminary data or no published evidence exists and we had no expert consensus. We also withheld recommendation when studies, no matter their LOE, showed conflicting data.

After answering the clinical question and providing recommendations for diagnostic and treatment interventions, we reviewed each current seizure protocol for the 33 agencies for consistency with the recommendations. The clinical protocols were reviewed during the month of June 2015. We deemed institutional review board approval not necessary for this review of publicly available research and clinical protocols.

## EVIDENCE REVIEW AND CURRENT RECOMMENDATIONS

### What is the appropriate prehospital treatment for a patient with a witnessed seizure who is not actively seizing?

Most seizures are brief and spontaneously resolve within one to two minutes.^5^ Patients with a seizure typically have transient hypoventilation that usually resolves quickly as long as their airway remains patent. Nonetheless, supplemental oxygen should be provided via nasal cannula or facemask, with suction and a nasopharyngeal airway readily available. Providers should be prepared to provide a jaw thrust and bag-valve mask ventilation as well to assist with spontaneous respirations if needed. Endotracheal intubation should be reserved for patients with respiratory failure after the seizure has stopped, and should be used only after other airway maneuvers and adjuncts have been attempted. Patients should be placed in a position of comfort that will also promote a patent airway, and which will minimize the risk of falls. The secondary survey should include an evaluation for signs of trauma. Initial management should also include a rapid assessment of blood glucose level. Although hypoglycemia is a relatively rare cause of seizures and has been demonstrated to be present in only 1.2% of patients with seizures, it is an inexpensive and rapid assessment tool that is widely available and hypoglycemia is readily reversible.^12^

There is conflicting opinion on the utility of routinely placing an intravenous line (IV) in patients who are not actively seizing. Since most patients will not require any medications once they are not actively seizing, there is not sufficient evidence to support routine IV access. The incidence of a second seizure within 72 hours has been reported to be approximately 6% and benzodiazepines administered intramuscularly (IM) are an effective treatment.^13^ Continuous pulse oximetry should be used to monitor oxygenation, and end-tidal CO_2_ monitoring, if available, should be used to detect hypoventilation in postictal patients until they have returned to their baseline mental status.

Patients who have had resolution of a seizure and have rapid return to their baseline sometimes refuse subsequent transport to the ED. In a 2016 retrospective study of patients who refused transport or were discharged at the scene by paramedics, improvement of symptoms in patients with a postictal state was a common reason for non-transport.^14^ In order to refuse additional care and/or transport to the hospital, a patient must have medical decision-making capacity to refuse care, which includes being alert and oriented, exhibiting no signs of intoxication, and demonstrating an understanding of the risks, benefits, and alternatives to refusing transport.^15^ Furthermore, the patient must be advised that paramedics will return if called again. This commonly involves the patient verbalizing an understanding of the medical condition and explaining the potential complications of refusing additional care and transport. A form documenting this encounter is typically signed by the patient and paramedic. EMS providers should report seizure activity as appropriate, and patients should be counseled not to drive due to the risk of additional seizures with the subsequent potential to injure both themselves and others.

Patients with first time or new-onset seizures should be strongly encouraged to accept transport to the ED since there are multiple life-threatening conditions that may be present. If refusing transport, these patients should be made aware of potential underlying medical conditions. Patients with known seizure disorders, such as epilepsy, commonly have breakthrough seizures due to medication non-adherence or under-dosing, sleep deprivation, infection, illicit substance use, or interactions with other medications. Despite seizure patients being more likely to be transported by EMS than other patients, a relatively high proportion still refuse ambulance transport.^16^ In a study of 2,619 pediatric calls for the chief complaint of seizure, 17% of parents/guardians refused transport to the ED. Rates of transport refusal may vary with geographic location, distance to the hospital, insurance status/cost of transport and individual frequency of complaint.

#### Current Prehospital Treatment Recommendation for a Patient with a Witnessed Seizure Who Is Not Actively Seizing

Level A Recommendation:

- None given.

Level B Recommendation:

- None given.

Level C Recommendation:

- No medications are recommended for a patient with a witnessed seizure who is not actively seizing.
- Post-seizure management should include supplemental oxygen by nasal cannula, continuous pulse oximetry and end-tidal CO_2,_ if available, with suction and nasopharyngeal airway immediately available. Bag-valve mask ventilation should be initiated for respiratory depression with endotracheal intubation reserved for prolonged respiratory failure.
- Patients with a witnessed seizure who are not actively seizing should be placed in a position of comfort, which also helps maintain a patent airway and minimize risk of falls.
- Blood glucose should be routinely checked in patients with suspected seizure if not returning to their baseline mental status.
- Routine placement of a prehospital IV may not be necessary for patients who are not actively seizing, and may be avoided if IV medications are not needed.

### What is the appropriate prehospital treatment for a patient who is actively seizing?

#### Choice of Benzodiazepine

Patients with prolonged or repeated convulsions lasting longer than five minutes are considered to be in SE and require immediate intervention.^17^ For EMS providers, calls dispatched for seizing patients who have ongoing seizures at the time of EMS evaluation suggests SE.^2^ Seizures lasting longer than 30 minutes have been shown to be less likely to terminate spontaneously and are associated with a higher mortality.^7^ Prolonged seizures cause both direct neuronal cellular injury as well as secondary complications such as impaired ventilation and aspiration, resulting in immediate neuronal loss followed by programmed cell death. Additionally, animal evidence indicates that resistance to benzodiazepines increases with longer seizure duration.^5^ As the time to effective treatment lengthens, the efficacy of first-line treatment with benzodiazepines decreases. Since earlier seizure cessation has been shown to improve outcomes and decrease cell death, rapid treatment and control of seizures has become a focus in the prehospital setting.^8^

With the knowledge that shorter time to seizure termination led to improved patient outcomes, prehospital providers began to initiate anticonvulsant therapy prior to hospital arrival. Since nearly all initial data were based on hospital and ED studies of seizures, research then began to focus on the safety and efficacy of prehospital EMS treatment with benzodiazepines. The Prehospital Treatment of Status Epilepticus (PHTSE) study was designed to determine whether benzodiazepines can be safely and effectively administered by paramedics to treat SE, whether prehospital treatment influences long-term patient outcome or ED disposition, and whether lorazepam, diazepam or placebo is superior for prehospital use in treating SE.^9^ The PHTSE study showed that SE was terminated in more patients who received IV lorazepam and diazepam than placebo (59.1% and 42.6% v 21.1%). Although there was no difference found between the lorazepam and diazepam groups, the study demonstrated that benzodiazepines could be successfully administered by EMS for the treatment of SE. This study also demonstrated that the termination of SE by the time of arrival to the ED correlated with better patient outcomes.

The PHTSE study showed that IV benzodiazepines (lorazepam and diazepam) are superior to placebo in terminating SE. Patients treated with benzodiazepines also had lower rates of respiratory compromise necessitating intubation, likely due to the shorter duration of seizures in the treatment groups.

To further investigate the administration of lorazepam for SE, a prospective, double-blind, randomized study of pediatric patients in an ED treated for SE compared IV diazepam to IV lorazepam.^18^ As part of the Pediatric Emergency Care Applied Research Network (PECARN), this study enrolled 273 pediatric patients with convulsive SE in 11 large academic hospitals in the U.S. No difference was found in the rate of cessation of seizures within 10 minutes (72.1% vs 72.9%), rate of recurrence within four hours (38.6% vs 39.2%) or rate of assisted ventilation (16.0% vs 17.6%) between the diazepam and lorazepam groups.

As midazolam became available for prehospital use, the Rapid Anticonvulsant Medication Prior to Arrival Trial (RAMPART) study was designed to compare IM midazolam to IV lorazepam. This landmark multicenter, double-blind, randomized, non-inferiority study of prehospital treatment of SE hypothesized that IM injection of a benzodiazepine would result in faster and more reliable medication administration, yielding improved seizure control prior to ED arrival.^19^ It used the Neurological Emergencies Treatment Trials (NETT) network to recruit adults and children estimated to weigh 13 kg or more. The findings of this study demonstrated that IM midazolam was as effective as IV lorazepam in terminating seizures without rescue therapy (73.4% vs 63.4%, p < 0.001 for non-inferiority and p < 0.001 for superiority), and was not associated with an increase in respiratory compromise or seizure recurrence. Additionally, the midazolam group had a lower rate of hospitalization. The RAMPART study concluded that although IV lorazepam had a quicker onset of action after administration, IM midazolam had a shorter time to administration since it did not require IV placement. Overall, however, there was no difference in time from medication box opening to seizure cessation between the IV and IM groups. Patients randomized to IM midazolam were more likely to have terminated seizures prior to ED arrival and were less likely to require hospital ward or intensive care unit (ICU) admission.^19^ Since adverse-event rates were similar between the two groups and lorazepam needs to be refrigerated, midazolam was deemed to be a safe and effective alternative for EMS treatment of SE. Additionally, studies have shown that midazolam has superior first-dose seizure suppression than diazepam.^20^

Non-benzodiazepine anticonvulsant medications have also been tested as both primary and secondary therapy for generalized convulsive SE. A prospective, randomized, double-blind study conducted at 16 Veterans Affairs medical centers and six affiliated university hospitals compared lorazepam, phenobarbital, phenytoin, and diazepam followed by phenytoin in 384 adult patients with SE.^21^

This study demonstrated that benzodiazepines (particularly lorazepam) were superior in stopping seizures. Lorazepam successfully terminated overt SE in 65% of 97 cases, similar to the results seen with phenobarbital and diazepam plus phenytoin, and superior to phenytoin alone. In a recent prehospital, randomized, double-blind, phase 3, placebo-controlled, superiority trial, levetiracetam was administered in addition to clonazepam for treatment of generalized convulsive SE. This treatment presented no advantage over clonazepam alone in the control of SE before arrival to the hospital.^22^

### Degradation of Drugs Issue

#### Which benzodiazepine is best suited to be stored in an ambulance environment?

EMS medications are frequently stored without temperature-control procedures, which may negatively impact the medication through degradation. Heat stability is an important factor in determining which benzodiazepine to deploy in an EMS system. Even in temperature-controlled environments, loss of power to mobile refrigerators and infrequently replaced cold packs in portable coolers may lead to inconsistent temperature regulation, especially in hotter climates.^23^ With temperature extremes known to occur inside vehicles, the choice of benzodiazepine carried by EMS should take into account medication performance after exposure to heat stress.

In an experimental pharmaco-stability study, diazepam and lorazepam solutions were stored for 210 days at refrigerated (4 to 10°C), ambient (15 to 30°C), and heated temperatures (37°C) to simulate real-world conditions.^24^ Drug concentration analysis was performed every 30 days to evaluate drug degradation. At ambient temperature, minimal (10%) concentration reduction was seen in diazepam after 30 days and lorazepam after 150 days. After 210 days, diazepam concentration reduction was 7% refrigerated, 15% ambient, and 25% heated. Lorazepam concentration reduction was 0% refrigerated, 10% ambient, and 75% heated. From these data, the authors concluded that diazepam had increased early degradation rates, but was more stable in the long term when heat stress was applied. Lorazepam exhibited better stability when refrigerated, but rapidly degraded when exposed to heat.

A subsequent study comparing midazolam to lorazepam demonstrated that midazolam remained stable at 60 days, but that lorazepam showed slight time- and temperature-dependent degradation.^25^ When midazolam and diazepam were compared to lorazepam in a follow-up study, both midazolam and lorazepam experienced minimal degradation throughout 120 days of EMS deployment in high-heat environments. Lorazepam, however, experienced significant degradation over 120 days and appeared especially sensitive to higher temperature exposure.^26^

### Route of Benzodiazepine

#### What is the preferred route of benzodiazepine administration in the treatment of status epilepticus?

Prehospital administration of benzodiazepines to terminate generalized convulsive seizures presents multiple safety considerations for both the treating provider and the patient. Involuntary muscle contractions during SE make prehospital IV placement more difficult to achieve and increase the chances of procedural complications. Providers may also be at increased risk for needle-stick injuries. Since time to medication and provider safety are priorities in treating SE, there have been studies of the preferred route of benzodiazepine administration. Traditionally, rectal (PR) or IV diazepam in children and IV diazepam in adults were considered to be the routes and drug of choice for prehospital medication administration.^17^,^27^ Newer studies, however, have focused on intramuscular and intranasal (IN) midazolam, which have been shown to have improved heat stability and may be preferred for ambulance storage, as discussed previously. To date, there are no data comparing IN to IM benzodiazepine administration. There have been a multitude of recent studies comparing intramuscular and intranasal midazolam to the traditional standard of IV diazepam or lorazepam; however, few studies exist that compare the novel administration routes of the same drug to each other. Additionally, much of the available research has focused on pediatric patients, with febrile seizures often included. Febrile pediatric seizures, therefore, will be discussed separately.

In a comparison of single dose PR vs IV diazepam for prehospital seizures in 31 pediatric patients, no difference was demonstrated in the rate of seizure cessation or recurrence of seizures prior to ED arrival.^27^ Although this study was a small, retrospective chart review, it also found no difference in prehospital or ED intubation rates.

There have been multiple case reports and descriptive studies demonstrating intramuscular midazolam as an effective therapy for SE; however, only one direct comparison of IV and IM midazolam was identified in the literature.^28^ In a retrospective chart review of 86 pediatric patients treated by EMS for prehospital seizures with either IV or IM midazolam, the IV group was found to have a significantly higher rate of clinical improvement, with no difference in admission rate.^29^ This study did not define their endpoint of “clinical improvement” as seizure cessation, however, and had nearly twice as many patients in the intravenous group (49 IV vs. 25 IM). Considering their findings, the authors concluded that “prehospital IV midazolam was an effective intervention for pediatric seizures.”

Despite the lack of research directly comparing IV with IM midazolam, the RAMPART study, previously described, demonstrated that IM midazolam was as effective as IV lorazepam in terminating seizures (73.4% vs 63.4). Although the IV group had a shorter time to seizure cessation after medication administration, the IM group had a shorter time to medication administration. This resulted in similar total times to seizure cessation between the groups. As previously noted, patients in the IM group were also less likely to be seizing upon arrival to the ED, regardless of the use or non-use of rescue therapy.^19^ This remained true when the pediatric patients in the study were considered separately, as described in a subsequent secondary analysis.^30^

With the advent of mucosal atomization devices, midazolam has also been administered by the IN route for seizure control. A study of 57 pediatric patients with SE compared IN mucosal atomized midazolam (IN-MAD) to rectal diazepam.^31^ The IN-MAD group, as compared to the rectal diazepam group, had shorter prehospital seizure duration and were less likely to have a seizure in the ED, undergo ED intubation, receive seizure medications for ongoing seizures in the ED, or be admitted to the hospital or pediatric ICU. Another study of 358 pediatric patients compared IN midazolam with rectal diazepam for the treatment of SE.^32^ There was no difference in time from medication administration to seizure cessation or complications between the diazepam or midazolam groups. Similarly, a prospective, randomized study of 45 pediatric patients comparing rectal diazepam to IN midazolam demonstrated that midazolam was more effective in terminating seizures within 10 minutes (87% vs 70%).^33^

In a unique longitudinal, crossover study of 124 seizure episodes in 21 adults with refractory SE, patient caregivers were able to administer rectal diazepam or IN midazolam at home.^34^ This study found no difference in successful treatment of seizure episodes between the diazepam group (89%) and the midazolam group (82%), and reported no severe adverse events in either arm.

While IN midazolam appears to be gaining popularity due to its ease and convenience of administration without needles, midazolam has also been delivered through a buccal route. In a study of adults living in a residential institution, buccal midazolam was found to be as safe and effective as rectal diazepam in terminating SE.^35^ Similar studies in children have also demonstrated that buccal midazolam is as effective as rectal diazepam in terminating convulsive seizures.^36^,^37^ Buccal midazolam has also been shown to be as effective as intravenous diazepam for seizure control in both partial and generalized convulsive seizures.^38^ While the time from medication administration to seizure control was less with IV diazepam, the time from initiation of treatment to seizure control was less with buccal midazolam.

### Dose of Benzodiazepine

#### What is the appropriate dose of intramuscular, intravenous, and intranasal benzodiazepine when treating status epilepticus?

Local protocols vary widely with regards to benzodiazepine dosing, with some using set dosages and others using a weight-based approach. The goal of either strategy is to maximize single-dose efficacy and minimize complications, such as respiratory depression. Factors that should be considered in choosing a medication dosage include the following: medication safety profile (i.e., toxic range); time to onset and peak level; duration of action; tissue distribution; and interactions with other medications. Although there have been relatively few studies that directly compare different dosages of the same medication delivered by the same route, much of the existing literature has used similar dosing ranges to demonstrate overall medication efficacy.

In a retrospective chart review of 288 pediatric patients with prehospital seizures, diazepam IV/rectal 0.2 to 0.5 mg/kg was compared to 0.05 to 0.1 mg/kg.^39^ Patients in the higher-dose group were more likely to require prehospital intubation and admission. Additionally, the IV diazepam group was more likely to require intubation than the rectal group. No difference was observed in the number of repeat doses or ED interventions.

A retrospective chart review of 93 pediatric patients treated by EMS for seizures received either diazepam IV 0.25 mg/kg or rectal 0.50 mg/kg prior to 1 January 2000, or midazolam IV 0.1 mg/kg or IM 0.2 mg/kg after the specified date.^40^ No difference was observed in rates of seizure cessation prior to ED arrival, seizure recurrence in ED, need for airway intervention, or admission rate. Significantly more patients initially administered IM midazolam required a second prehospital dose as well as additional benzodiazepines in the ED, compared to the IV midazolam group.

As discussed previously, the RAMPART study compared IM midazolam 10 mg to IV lorazepam 4 mg in adults and children weighing more than 40 kg, and IM midazolam 5 mg to IV lorazepam 2 mg in children with an estimated weight of 13 to 40 kg.^19^ Pooling the high- and low-dosage data together, this study demonstrated that IM midazolam (448 patients) was as effective as IV lorazepam (445 patients) in terminating seizures without rescue therapy (73.4% vs 63.4%), and showed no difference in frequency of endotracheal intubation or seizure recurrence. Of note, both midazolam and lorazepam groups consisted primarily of high dosage administrations, with 386 (86% of midazolam and 87% of lorazepam) high dose administrations in both arms.

In a prospective, randomized, blinded comparison of IV diazepam 0.2 mg/kg to IN midazolam 0.2 mg/kg administered to 70 pediatric patients with acute seizures, no difference was observed in seizure cessation within 10 minutes.^41^ Additionally, the time from seizure onset to treatment was shorter in the midazolam group, although the time from seizure onset to cessation was shorter in diazepam group. In a similar study, which used the same 0.2 mg/kg dose of IN midazolam but a higher, 0.3 mg/kg, dose of IV diazepam, there was also no difference in the rate of seizure termination between the groups, and the time from arrival at hospital to seizure cessation was shorter in the midazolam group.^42^

A retrospective, observational study of 57 pediatric patients comparing IN midazolam 0.2 mg/kg (max dose 10 mg) to PR diazepam 0.3–0.5 mg/kg (max 20 mg) found that the midazolam group had shorter prehospital seizure duration, were less likely to have seizure recurrence, undergo intubation, receive seizure medications for ongoing seizures in the ED, or be admitted to hospital.^31^

### Appropriate Order of Benzodiazepine and Glucose Measurement

#### Should paramedics measure a glucose level in those patients with a history of a seizure prior to administering a benzodiazepine?

Most EMS protocols require blood glucose testing during the evaluation of SE. There has been little agreement on when this testing should be performed since hypoglycemia can manifest as seizures, but checking blood glucose may delay the administration of benzodiazepines.^12^ While some protocols require checking blood glucose prior to the administration of benzodiazepines, others leave the timing to the discretion of the treating paramedic.^4^,^12^

In a retrospective observational study of 53,505 EMS calls for seizure where blood glucose was measured, hypoglycemia was present in 638 (1.2%) patients with seizures.^12^ Seizing patients were treated with benzodiazepine in 8.3% and with glucose in 1.3% of patients. Obtaining a blood glucose measurement was associated with a 5.9-minute delay in benzodiazepine administration compared to patients who had no blood glucose tested, and 2.1-minute delay compared to patients who had glucose testing performed after benzodiazepine administration. Since rates of hypoglycemia were very low in patients treated by EMS for seizure, the study concluded that glucose testing prior to benzodiazepine administration was not supported.

### IV Placement in Active Seizure Patient

#### Should paramedics place an IV in those patients who are actively seizing?

Despite the success of IM and IN benzodiazepines in terminating SE prior to ED arrival, there remains a significant number of patients who will require additional anticonvulsant therapy. If IM or IN therapies are not successful in terminating active seizures, IV benzodiazepines may be necessary. Additionally, since the rate of respiratory failure requiring intubation increases with the length of seizure activity, it is likely that IV placement will be needed if initial IM or IN treatment fails to terminate seizure activity.^9^,^17^

In a secondary analysis of the RAMPART study, 218 patients (21%) required endotracheal intubation for respiratory depression, altered mental status, or recurrent seizures after initial termination.^43^ Fourteen (6.4%) endotracheal intubations were performed in the prehospital setting and 204 (93.6%) occurred in the hospital. Endotracheal intubation occurred less frequently in patients younger than 50 years of age and in women compared to men. Additionally, mortality was higher in patients undergoing late intubation (greater than 30 minutes after ED arrival). This analysis demonstrates that despite prehospital treatment of SE, there remains a substantial proportion of patients who require advanced airway management and additional therapy. Although an IV can be placed after the patient’s arrival to the ED, EMS can shorten the time to definitive treatment by placement of an IV after prehospital therapy has been initiated.

#### Current Prehospital Treatment Recommendation for the Patient in Status Epilepticus

Level A Recommendation:

- IM injection of midazolam should be the first-line EMS treatment of the patient in SE without an established intravenous line.
- The suggested initial dose of IM midazolam is 0.2 mg/kg, with a max of 10 mg in adults and children greater than 40 kg.
- The suggested initial dose of IN and buccal midazolam is 0.2 mg/kg, with a max of 10 mg in adults and children greater than 40 kg.
- The suggested initial dose of IV midazolam and lorazepam is 0.1 mg/kg, with a max dose of 4 mg in adults and children greater than 40 kg.

Level B Recommendation:

- Midazolam is the preferred benzodiazepine when stored in an ambulance and potentially exposed to heat stress.
- If IM injection is contraindicated, IN or buccal midazolam should be used as a second-line therapy in SE.
- If midazolam is not available or contraindicated, IV lorazepam should be used as an alternative therapy in SE.

Level C Recommendation:

- Blood glucose should be routinely checked in patients with SE only after benzodiazepine administration.
- Routine placement of a prehospital IV is recommended, after initial dose of IM or IN benzodiazepine has been administered.
- When administering IN midazolam, a highly concentrated solution of 5 mg/1 ml is preferred to minimize volume of medication delivered.
- IV and rectal diazepam is no longer recommended for the routine initial treatment of SE in the prehospital environment.
- Post-seizure airway management should include supplemental oxygen by nasal cannula, continuous pulse oximetry and end-tidal CO_2_ if available, with suction, nasopharyngeal airway, bag-valve mask and endotracheal intubation immediately available for signs of respiratory failure. Monitoring of the airway is particularly important for those patients who receive treatment with benzodiazepines.
- Caution should be used when administering benzodiazepines to the same patient by both IV and IM routes since absorption differs by route.

### Pediatric Febrile Seizures

#### What is the appropriate prehospital treatment for a pediatric patient with febrile status epilepticus?

Among children with seizures, febrile seizures are the most common type, accounting for almost one third of all pediatric seizures in the ED.^16^ Up to 10% of children with febrile seizures develop febrile status epilepticus (FSE).^44^ This subset of pediatric seizures accounts for 25% of all childhood SE and more than two thirds of SE in the second year of life.^44^

Since both febrile and afebrile pediatric SE are thought to cause similar neuronal damage and respiratory complications, they have traditionally been treated similarly in the prehospital environment. There has been little research directly comparing the two groups, and much of the prehospital and ED seizure research to date has included both febrile and afebrile patients together. Benzodiazepines remain the mainstay of treatment for any generalized convulsion, and treatment of pediatric FSE by EMS has largely focused on rapid and minimally invasive routes of medication administration.

In recent years, efforts to improve the administration of anticonvulsant drugs through rapid non-invasive routes have become common in prehospital care. This is especially pertinent to the pediatric population, which may have increased difficulty with IV line placement. Findings from a study of 28 children in the prehospital setting showed that buccal midazolam was as safe and effective as rectal diazepam (75% in midazolam group vs 59% in diazepam group) in terminating seizures.^36^ A subsequent randomized controlled trial found buccal midazolam to be superior to rectal diazepam for children actively seizing at the time of presentation to the ED.^37^ Additionally, there was no difference in rates of respiratory compromise between the groups; however, the diazepam group had higher rates of seizure recurrence after initial cessation. As discussed previously, a randomized controlled study of 126 patients comparing buccal midazolam to IV diazepam also found no difference in overall rates of seizure control, but did demonstrate faster time from initiation of treatment to seizure cessation in the buccal midazolam group.^38^

Results from a prospective, randomized study of pediatric patients with prolonged febrile seizures showed that IN midazolam was as effective as IV diazepam for seizure control.^46^ In this study, 44 children were randomized to receive IN midazolam 0.2 mg/kg or IV diazepam 0.3 mg/kg for febrile seizures lasting at least 10 minutes. The convulsions were determined to be febrile seizures in a retrospective chart review. The time from arrival at the hospital to seizure control was faster in the midazolam group, and no difference as observed in rates of respiratory depression or bradycardia between the groups.

Despite the relative safety and efficacy of benzodiazepine administration for prehospital seizures, there remains a significant proportion of patients who do not receive medication prior to arrival at the ED. In one study of pediatric patients with SE in the U.S., 63% of patients did not receive any anticonvulsant medication prior to hospital arrival.^47^ Although some of these patients were enrolled prior to the results of the RAMPART study, this serves as a reminder of the ongoing need for improvement in our EMS systems.

#### Current Prehospital Treatment Recommendation for Febrile Seizures

Level A Recommendation:

- IM injection of midazolam should be the first line EMS treatment of the actively seizing febrile pediatric patient.
- The suggested initial dose of IM midazolam is 0.2 mg/kg, with a max of 10 mg in children greater than 40 kg.
- The suggested initial dose of IN and buccal midazolam is 0.2 mg/kg, with a maximum of 10 mg in children greater than 40 kg.
- The suggested initial dose of IV midazolam and lorazepam is 0.1 mg/kg, with a max dose of 4 mg in children greater than 40 kg.

Level B Recommendation:

- If IM injection is contraindicated, IN or buccal midazolam should be used as a second-line therapy in the actively seizing febrile pediatric patient.
- If midazolam is not available or contraindicated, IV lorazepam should be used as an alternative therapy in the actively seizing febrile pediatric patient.

Level C Recommendation:

- Pediatric FSE should be treated with the same treatment considerations as afebrile pediatric SE.
- Febrile pediatric patients who are no longer actively seizing should be transported to the ED without any anticonvulsant medication administration.
- Cooling measures should be initiated after benzodiazepine administration, as long as they do not interfere with routine care.
- Blood glucose should be routinely checked in pediatric patients with SE only after benzodiazepine administration and if the patient does not show progressive improvement in mental status.

### Eclampsia

#### What is the appropriate prehospital treatment for a mid- to late-term pregnant patient who is actively seizing?

Eclampsia, characterized by seizure activity after the 20^th^ week of pregnancy, is a rare but significant cause of mortality worldwide.^48^ Although eclampsia may occur alongside existing pre-eclampsia, characterized by hypertension and proteinuria during pregnancy, it may also occur independently. In the U.S., approximately 15% of obstetric deaths are associated with pre-eclampsia or eclampsia. The incidence of eclampsia worldwide approaches 150,000 cases annually, with approximately 0.92 cases per 1,000 deliveries in the U.S.^49^ In a 2016 study of prehospital EMS activations for pregnancy-related emergencies, however, eclampsia made up only 0.5% (19/4,096) of calls involving a pregnant or post-partum patient.^50^ Eclamptic seizures may occur during the second half of pregnancy, during labor, or after childbirth. Although the underlying cause and pathophysiology of eclampsia is not completely understood, risk factors that put patients at greater risk include the following: family history of eclampsia; reduced prenatal care; age less than 20 years; multiple prior pregnancies (≥4); and ≥ 2 symptoms including headache, abdominal pain, hyper-reflexia, or visual disturbances. Eclampsia has historically been treated with an anticonvulsant to control acute seizures as well as maintenance anticonvulsant therapy until delivery can be accomplished.

There remains controversy over whether magnesium sulfate is a true anticonvulsant and should be used to treat active seizures, or is instead primarily useful in preventing additional seizures.^51^ Magnesium sulfate has been hypothesized to have central nervous system anticonvulsant effects through various mechanisms including NMDA-receptor down-regulation and blood-brain barrier protection, based on in-vitro and animal models.^52^–^55^ Since few large-scale studies comparing treatments for eclampsia have been conducted, the Cochrane Collaboration published a systematic review of seven such randomized trials in 2010.^48^ It should be noted, however, that 65% of the data (910/1,396 patients) came from a single study: “Collaborative Eclampsia Trial.” An earlier study had found a trend towards improved outcomes in patients with eclampsia treated with magnesium sulfate compared to diazepam; however, the study was not powered to detect a difference in seizure recurrence.^56^ The Cochrane Review, in contrast, demonstrated fewer recurrent seizures after treatment with magnesium sulfate compared to both diazepam and phenytoin.^48^,^57^ Although there was no difference in neonatal or perinatal mortality, fewer babies had Apgar scores less than seven at one minute or at five minutes in the magnesium group vs diazepam group.^48^ This remains the most conclusive evidence to date, and has been universally adopted as the standard of care in treatment of eclampsia.^58^ In 2002, the American College of Obstetricians and Gynecologists published Level A recommendations for the treatment of eclampsia with magnesium sulfate IV or IM, typically with a 4 – 6 g initial IV loading dose followed by 2 g per hour infusion.^59^ While the ideal prehospital treatment of eclampsia remains somewhat unclear, seizure control with an anticonvulsant agent and airway management are of paramount importance.

#### Current Prehospital Treatment Recommendation for Eclampsia

Level A Recommendation:

- Actively seizing patients who are known to be pregnant or postpartum should be treated with magnesium sulfate 4 to 6 g IV.

Level B Recommendation:

- None given.

Level C Recommendation:

- If an IV cannot be established quickly, an initial dose of magnesium sulfate 10 g IM may be administered as an alternative (with 5 g administered IM in each buttock).
- IM or IV benzodiazepines should be considered in the treatment of refractory seizures in the pregnant or postpartum patient unresponsive to magnesium sulfate.
- Blood glucose should be routinely checked in patients with suspected eclampsia.
- Airway management should include supplemental oxygen, bag-valve mask ventilation, and endotracheal intubation immediately available for respiratory failure.
- Patients should be placed in a position of comfort, which also helps maintain a patent airway and minimize risk of falls. If hypotension is present, the patient should be placed in the left lateral decubitus position, as tolerated.

## RESULTS

All 33 agencies have protocols, which were identified and reviewed for consistency with the recommendations made by EMDAC for prehospital seizure management (Tables 1 and 2). Every agency has a protocol relating to the treatment of seizures, although these protocols vary significantly. Multiple drugs, dosages, routes of administration, re-dosing instructions, and requirement for blood glucose testing prior to medication delivery were found. Examples of suggested language for protocol development that the committee felt was most consistent with the recommendations were taken from the agency protocols.

### Witnessed Seizure Not Actively Seizing

Few of the seizure protocols in California specifically mention the treatment of patients who are not actively seizing. Routine care, including airway management and evaluation for underlying causes, are typically recommended.

### Choice of Benzodiazepine in Actively Seizing Patient

The overwhelming benzodiazepine of choice in California for patients who are actively seizing is midazolam. There was variation in dosing of the IM, IN, and IV/IO routes described in each protocol. One EMS agency uses lorazepam as a first-line agent with midazolam available as a second-line therapy. Two rural California agencies have special use of diazepam for EMT-IIs in their counties. Several agencies provide the option of diazepam and/or lorazepam as possible substitutes in the case of drug shortages. All agencies have protocols for giving IV and IM benzodiazepines and 76% (25/33) have protocols for IN benzodiazepines.

### Dose of Benzodiazepine in Actively Seizing Patient

IM midazolam dosages ranged from 2 to 10 mg per single adult dose, 2 to 8 mg per single pediatric dose, and 0.1 to 0.2 mg/kg as a weight-based dose. IN midazolam dosages ranged from 2 to 10 mg per single adult or pediatric dose, and 0.1 to 0.2 mg/kg as a weight-based dose. IV/IO midazolam dosages ranged from 1 to 6 mg per single adult dose, 1 to 5 mg per single pediatric dose, and 0.05 to 0.1 mg/kg as a weight-based dose.

### Order of Benzodiazepine and Glucose Measurement in Actively Seizing Patient

Blood glucose testing *prior* to benzodiazepine administration is required by 61% (20/33) of agencies for adult patients and 76% (25/33) for pediatric patients. Nine percent (3/33) of agencies recommend checking blood glucose prior to benzodiazepine administration if hypoglycemia is suspected or there is a known history of diabetes mellitus. This has been identified as an area for improvement in our clinical protocols for the seizing patient.

### Pediatric Febrile Seizures

Sixty-seven percent (22/33) of agencies specifically address the treatment of febrile seizures. One agency has a protocol for administering acetaminophen or ibuprofen to patients with febrile seizures. Fifty-eight percent (19/33) of agencies have a protocol for passive and/or active cooling *prior* to administration of benzodiazepines.

### Eclampsia

Eclampsia is specifically addressed by 85% (28/33) of agencies. Forty-two percent (14/33) of agencies have a protocol for administering magnesium sulfate to patients with eclampsia, with dosages ranging from 2 to 6mg IV and 58% (19/33) allow benzodiazepines to be administered.

## DISCUSSION

The adult and pediatric seizure protocols varied greatly in content and structure between local EMS agencies in the State of California. These government agencies consist of either a county or region that develops a system of care that includes first responders, ambulance transporters, and specialty receiving facilities. These systems reflect the needs and demographics of that county or region and operate under one set of medical control policies. A similar variation among protocols was seen in a recent study on statewide EMS protocols.^60^ In 2014, the National Association of EMS Officials published model EMS guidelines that could be used to decrease this variability.

## LIMITATIONS

This study is limited by the fact that only the protocols of one state were evaluated and might not be generalizable to other geographic areas. There are always inherent biases involved in the analysis of the available evidence and the synthesis into recommendations. Our clinical questions could not always be answered by specific prehospital research. When appropriate, research that was completed in a hospital setting was extrapolated to answer our question.

## CONCLUSION

Protocols for adult and pediatric seizures, including eclampsia and febrile seizures, vary widely across the State of California. The evidence-based recommendations that we present for the prehospital diagnosis and treatment of this condition may be useful for EMS medical directors tasked with creating and revising these protocols.

## Supplementary Information



## Figures and Tables

**Table 1:** Adult Seizure protocols.

		Midazolam			
			
LEMSA	Blood glucosre prior To BZD	IN	IM	IV/IO	Eclampsia
Alameda County	No	5 mg	0.1 mg/kg to max 6mg, give full dose IM	0.1 mg/kg in 1–2mg increments (no specified frequency, max single dose 6mg)	Midazolam (follow seizure protocol)
Central California EMS	No	0.1 mg/kg IN to max of 4mg	0.1 mg/kg to max 4mg × 1 only	0.05 mg/kg to max 2mg, repeat × 1 at 10min	Midazolam, then Mag 5g IV over 20 min
Coastal Valleys EMS	Yes	5mg MR ×1 at 10min	5 mg MR ×1 at 10min	2mg Q5min PRN, net max 10mg by all routes	Midazolam 2mg, if transport > 1hr, optional Magnesium 2g followed by drip
Contra Costa County	Yes	N/A	0.1 mg/kg to max 5mg	1mg, titrate in 1–2mg increments to max 5mg	----
El Dorado County	Yes	5mg MR at 5min	5mg	2.5mg MR at 5min	Mag 6g IV over 1–2min
Imperial County	Yes	0.2 mg/kg to max 10mg, repeat × 1 per BH	0.2 mg/kg to max 10mg, repeat × 1 at 10min	0.1 mg/kg to max 5mg, repeat × 1 at 10min	Midazolam per SZ Protocol w/all repeat doses per BH
Inland Counties EMA	Yes	2.5mg MR at 5min (max 3 doses all routes)	5mg MR at 10min (max 3 doses all routes)	2.5mg MR at 5min (max 3 doses all routes)	Mag 4g over 3–4min, gtt at 0.6 g/hr to 2g
Kern County	Yes	1st line: lorazepam 2mg IV/IO/IM Q10min to max 4mg	2nd line: midazolam 1mg IV/IN Q2min PRN to max 5mg w/BHO	3rd line: diazepam 5mg IV Q10min PRN to max 30mg w/BHO	1st line: Mag 2–4g IV
					2nd line: diazepam 5mg IV Q10min PRN to max 30mg
Los Angeles County	Yes	5mg MR ×1 at 5min	5mg MR ×1 at 5min	2–5mg MR ×1 at 5min, net max 10mg by all routes	DO NOT delay transport for treatment
Marin County	Yes	5mg	0.1 mg/kg MR × 1 at 10min	1mg Q3min PRN to max 0.05 mg/kg	Midazolam, NTG for HTN
Merced County	No	N/A	0.2 mg/kg to max 8mg, may NOT repeat	0.1 mg/kg to max 4mg, MR (no specified frequency or net max)	Mag 2g IV over 2 min
Monterey County	No	2mg MR ×1	2mg MR ×1	1–2mg Q5min PRN to max 4mg	----
Mountain Valley EMS	Yes	N/A	5mg MR ×1 at 10min	2mg then titrate 1mg increments to max 6mg (no specified frequency)	----
Napa County	Yes	5mg × 1 only (MR ×1 at 5min for eclampsia)	0.1 mg/kg to max 6mg (max 5mg fpr eclampsia)	1mg then titrate 1mg increments to max 6mg (no specified frequency)	Midazolam, NTG for HTN (need base contact)
Nor-Cal EMS	Yes	0.2 mg/kg to max 10mg, MR ×1 to 20mg w/BHPO	N/A	2mg Q2min PRN to max 10mg, max 20mg w/BHPO	Mag 4g IV over 20min followed by gtt at 2 g/hr
EMT-II		Diazepam 2.5–10mg IVP over 2min, if no access 10mg IM. MR ×1 to max 20mg IV/IM/IO w/BHPO			
North Coast EMS	Yes	5mg w/10mg max	5mg (0.07 mg/kg)	1–2.5mg repeat PRN to max 0.1 mg/kg not to exceed 10mg	Mag 4g IV over 20min followed by gtt at 1–2 g/hr
EMT-II		Diazepam 2.5–20mg IVP titrated in 2.5mg increments to max 40mg. 5–10mg IM			
Orange County	No	5mg MR ×1 at 3 min	5mg MR ×1 at 3 min	N/A	Midazolam
Riverside County	Yes	2.5mg MR ×1 (all dosages per chart)	5mg MR × 1	2.5mg MR × 1	Mag 5g IV over 10min or 2.5g IM × 2 divided doses
		May substitute lorazepam 5mg IM, 2.5mg IN/IV/IO OR diazepam 5mg IM, 2.5mg IV/IO. MR ×1			
Sacramento County	Yes	0.1 mg/kg to max 6mg	0.1 mg/kg to max 6mg	0.1 mg/kg to max 6mg in 2mg increments	----
		May substitute diazepam (in context of midazolam shortage) 5–10mg IV/IO, 10mg IM, MR IM ×1			
San Benito County	Yes	0.1 mg/kg to max 5mg, additional per BH	0.2 mg/kg to max 10mg, additional per BH	0.1 mg/kg to max 5mg, additional per BH	Midazolam (follow seizure protocol)
San Diego County	No	Midazolam IN/IM/IV/IO to max 5mg, MR × 1 at 10min to max 10mg (no specified increment or interval)			Midazolam IN/IM/IV/IO to max 5mg, MR ×1 at 10min to max 10mg
San Francisco County	No	5mg ×1	5mg ×1	2.5mg MR at 5min, max dose 5mg	Magnesium sulfate
San Joaquin County	No	4mg MR Q5min to max 10	2 or 4mg(?) MR Q5min to max 10mg	2mg MR Q5min to max 10mg	Mag 2g IV over 3–5min after base hospital contact
San Luis Obispo County	No	5mg MR ×1 at 10min	0.1 mg/kg to max 5mg MR ×1 at 10min	1–2mg MR ×1 at 10min	Midazolam (follow seizure protocol)
San Mateo County	No	N/A	1–2mg Q5min PRN to max 10mg	1–2mg Q5min PRN to max 10mg	Midazolam IV/IM 1–2mg Q5min PRN to max 10mg
Santa Barbara County	Yes if known h/o DM	N/A	0.1 mg/kg to max 5mg	1mg Q2min PRN to max 5mg	Mag 2g IV over 5 min, MUST repeat ×1, midazolam if persistent seizures > 2 min
Santa Clara County	Yes	5mg	0.1 mg/kg to max 5mg	2mg Q2min PRN to max 5mg	midazolam (follow seizure protocol)
		2nd choice: diazepam 5–10mg IV, MR at 5min to max 15mg	3rd choice: lorazepam 1–2mg IV Q10min PRN to max 8mg		
Santa Cruz County	Yes	N/A	0.2 mg/kg to max 10mg	0.1 mg/kg to max 5mg	Midazolam (follow seizure protocol)
Sierra Sacramento Valley EMS	Yes	0.2 mg/kg to max 8mg MR ×1 at 5min	0.2 mg/kg to max 8mg MR ×1 at 5min	0.1 mg/kg to max 4mg MR ×1 at 5min	Midazolam (follow seizure protocol)
Solano County	Yes	N/A	4mg	2mg	Midazolam (follow seizure protocol)
Tuolumne County	No	1–2mg Q3min PRN to max to max 10mg by all routes	2mg Q10min to max 10mg by all routes	1–2mg Q3min PRN to max to max 10mg by all routes	Midazolam (follow seizure protocol), THEN Magnesium 2g IV after base contact
Ventura County	Yes	N/A	0.1 mg/kg to max 5mg	2mg repeat 1mg Q2min PRN to max 5mg	Mag 2g IV over 5min, MUST repeat ×1, midazolam if persistent seizures > 2 min
Yolo County	No	5mg may NOT repeat	0.1 mg/kg to max 6mg may NOT repeat	2mg repeat 1mg Q5min PRN to max 6mg	----

*BZD,* benzodiazepine; *IN,* intranasal; *IM*, intramuscular; *IV/IO,* intravenous/interosseous; *mg,* milligram; *mg/kg,* milligram per kilogram; *MAX,* maximum; *MAG,* magnesium; *MR,* may repeat; *PRN,* as needed; *Q,* every; *BH,* base hospital; *MIN,* minute; *SZ*, seizure; *GTT,* drops; *BHO,* base hospital; *NTG,* nitroglycerin; *HTN,* hypertension; *BHPO*, base hospital physician; *EMT-II,* emergency medical technician; *DM,* diabetes mellitus; *CBG,* capillary blood glucose; *PR,* per rectum; *SMC,* San Mateo County

**Table 2:** Pediatric seizure protocols.

		Midazolam			
			
LEMSA	CBG Prior to 1st dose BZD	IN	IM	IV/IO	Febrile
Alameda County	No after benzos	0.2 mg/kg	0.1 mg/kg to max 5mg, give full dose IM	0.1 mg/kg in 1–2mg increments (no specified frequency, max single dose 5mg)	cooling after benzos
Central California EMS	No	0.1 mg/kg IN to max of 4mg	0.1 mg/kg to max 4mg × 1 only	0.05 mg/kg to max 2mg, repeat × 1 at 10min	----
Coastal Valleys EMS	Yes	0.1 mg/kg MR ×1 at 5min to max 5mg	0.1 mg/kg MR ×1 at 5min to max 5mg	0.1 mg/kg MR ×1 at 5min to max 5mg	cool if febrile
Contra Costa County	Yes	N/A	0.1 mg/kg to max 5mg	1mg increments to max 0.1 mg/kg	remove clothing to “address cooling”
El Dorado County	Yes	0.1 mg/kg max total 3mg	0.1 mg/kg max total 3mg	0.1 mg/kg max total 3mg	external cooling with water, Tylenol or Ibuprofen
Imperial County	Yes	0.2 mg/kg (not listed in peds drug guide)	0.2 mg/kg in max 1–2mL increments	0.1 mg/kg to max 5mg	----
Inland Counties EMA	Yes	0.2 mg/kg to max 5mg MR at 10min	0.2 mg/kg to max 5mg MR at 10min	0.1 mg/kg to max 2.5mg MR at 5min	cooling before benzos
Kern County	Yes	1st line: midazolam 0.2mg/kg IM MR at 15min, IV/IO/IN to max 6mg	2nd line: lorazepam 0.1 mg/kg IV/IM/IO MR at 5min to max 0.2 mg/kg not to exceed 4mg	3rd line: diazepam 0.3 mg/kg IV/IO MR Q25min to max 5mg, 0.5 mg/kg PR to max 10mg	----
Los Angeles County	Yes	0.1 mg/kg MR ×1 at 5min to max 5mg by all routes	0.1 mg/kg MR ×1 at 5min to max 5mg by all routes	0.1 mg/kg MR ×1 at 5min to max 5mg by all routes	cooling before benzos
Marin County	Yes	0.2 mg/kg to max 5mg	0.1 mg/kg MR × 1 at 10min	0.05 mg/kg (max 1mg/dose) Q3min PRN to max 5mg	“treat fever prior to benzos”
Merced County	Yes	N/A	0.2 mg/kg to max 8mg, may NOT repeat	0.1 mg/kg to max 4mg, MR ×1 at 15min	----
Monterey County	Yes	0.2 mg/kg to max 2mg, repeat by BHO only	0.2 mg/kg to max 2mg, repeat by BHO only	0.1 mg/kg to max 2mg, repeat by BHO only	External cooling with water, fans
Mountain Valley EMS	Yes	N/A	0.2 mg/kg to max 5mg MR ×1 at 10min	0.1 mg/kg to max 5mg MR ×1 at 10min	----
Napa County	Yes	0.2 mg/kg to max 5mg, may NOT repeat	0.1 mg/kg to max 5mg, may NOT repeat	1mg then titrate 1mg increments to max 5mg (no specified frequency)	----
Nor-Cal EMS	Yes	0.2 mg/kg to max 10mg, MR ×1 to 10mg w/BHPO; PR 0.3 mg/kg MR ×1*	N/A	0.1 mg/kg * *patient weight > 20kg, if less requires BHPO	----
EMT-II		Diazepam 0.2 mg/kg IM/IV/IO MR ×1 to max 10mg w/BHPO			
North Coast EMS	Yes	0.1 mg/kg	0.1 mg/kg subsequent up to 0.4 mg/kg to max 5mg, net max 10mg	0.05 mg/kg max 5mg, net max 10mg	Cool with moist towels
EMT-II		Diazepam 0.1–0.3 mg/kg IVP or 0.5 mg/kg PR to max 20mg			
Orange County	No	0.1 mg/kg to max 5mg, MR ×1 at 3mins	0.1 mg/kg to max 5mg, MR ×1 at 3mins	N/A	----
Riverside County	Yes	(Per Broselow)	(Per Broselow)	(Per Broselow)	cooling measures before benzos
		May substitute lorazepam or diazepam			
Sacramento County	Yes	0.2 mg/kg to max 6mg	0.1 mg/kg to max 4mg	0.1 mg/kg to max 4mg in 1–2mg increments	undress pt for cooling
		May substitute diazepam (in context of midazolam shortage) 0.1 mg/kg IM/IV/IO to max 5mg, MR IM ×1			
San Benito County	Yes	0.1 mg/kg to max 3mg, additional per BH	0.2 mg/kg to max 3mg, additional per BH	0.1 mg/kg to max 3mg, additional per BH	----
San Diego County	No	(Per Broselow)	(Per Broselow)	(Per Broselow)	“Versed not required for simple febrile seizures”
San Francisco County	Yes	0.2 mg/kg total max dose 2mg*	0.1 mg/kg total max dose 2mg*	0.1 mg/kg total max dose 2mg* *may substitute Diastat rectal gel if available	“Cooling measures if fever present”
San Joaquin County	Yes	0.2 mg/kg to max 5mg	0.1 mg/kg to max 5mg	0.1 mg/kg to max 5mg	“initiate cooling if febrile”
San Luis Obispo County	No	0.1 mg/kg to max 5mg all routes MR ×1 at 10min	0.1 mg/kg to max 5mg all routes MR ×1 at 10min	0.1 mg/kg to max 5mg all routes MR ×1 at 10min	----
San Mateo County	Yes	N/A	(Per SMC reference card to max 5mg)	(Per SMC reference card to max 5mg)	cooling before benzos
Santa Barbara County	If known h/o DM	N/A	0.1 mg/kg to max 5mg	0.1 mg/kg to max 1mg	passive cooling measures
Santa Clara County	Yes	0.1 mg/kg to max 5mg	0.1 mg/kg to max 5mg	0.1 mg/kg to max 5mg	remove clothing to address cooling
		2nd choice: diazepam IV 0.25 mg/kg to max 5mg age < 5y, max 10mg > 5y; PR 0.5 mg/kg to max 10mg; 3rd choice: lorazepam 0.1 mg/kg to max 2mg MR ×1 at 5–10min to net max 4mg			
Santa Cruz County	Yes	N/A	0.2 mg/kg to max 3mg total	0.1 mg/kg to max 3mg total	remove clothing to address cooling
Sierra Sacramento Valley EMS	Yes	0.2 mg/kg to max 8mg MR ×1 at 5min	0.2 mg/kg to max 8mg MR ×1 at 5min	0.1 mg/kg in 1–2mg increments to max 4mg MR ×1 at 5min	remove clothing to address cooling
Solano County	Yes	N/A	0.2 mg/kg to max 4mg	0.1 mg/kg titrated in 1mg increments Q3min to max 5mg	cooling if febrile
Tuolumne County	No	0.2 mg/kg to max 2mg	0.1 mg/kg to max 1mg	0.1 mg/kg to max 1mg	----
Ventura County	Yes	N/A	0.1 mg/kg to max 5mg	N/A	passive cooling measures
Yolo County	No	0.2 mg/kg to max 8mg MR ×1 at 5min with BHO	0.2 mg/kg to max 8mg MR ×1 at 5min with BHO	0.1 mg/kg in 1–2mg increments repeat Q5min, max single dose 4mg	cooling only AFTER seizures stop/controlled

*BZD,* benzodiazepine; *IN,* intranasal; *IM*, intramuscular; *IV/IO,* intravenous/interosseous; *mg,* milligram; *mg/kg,* milligram per kilogram; *MAX,* maximum; *MAG,* magnesium; *MR,* may repeat; *PRN,* as needed; *Q,* every; *BH,* base hospital; *MIN,* minute; *SZ*, seizure; *GTT,* drops; *BHO,* base hospital; *NTG,* nitroglycerin; *HTN,* hypertension; *BHPO*, base hospital physician; *EMT-II,* emergency medical technician; *DM,* diabetes mellitus; *CBG,* capillary blood glucose; *PR,* per rectum; *SMC,* San Mateo County
