# Supplementary material for: Prehospital Care for the Adult and Pediatric Seizure Patient: Current Evidence-based Recommendations
Source: West J Emerg Med. 2017 Mar 3;18(3):419–36. doi: 10.5811/westjem.2016.12.32066 (PMC5391892; doi:10.5811/westjem.2016.12.32066)
Supplement: Supplementary file 1 [file wjem-18-419-s001.pdf]

Evidentiary Table: Blood Glucose Testing

Study	LOE	Study Design	Methods and Outcomes	Results	Limitations
Beskind 2014[1]	III	Retrospective observational study	53,505 EMS calls for seizure with a blood glucose measurement recorded were reviewed from a national ambulance service database of 140 ALS systems. The rates of identified hypoglycemia (blood glucose < 60 mg/dL) and subsequent treatment were tabulated. Rates of benzodiazepine administration and time to administration were compared.	Hypoglycemia was present in 638 (1.2%) of patients with seizures and 487 (0.9%) were treated with glucose. Overall treatment of seizing patients with benzodiazepine occurred in 6,389 (8.3%) of and with glucose in 975 (1.3%) patients. Obtaining a blood glucose was associated with a 5.9 minute and 2.1 minute delay in benzodiazepine administration in patients with no CBG or CBG after benzo, respectively.	Retrospective study, no outcome data on patients treated in ED/inpatient.

Evidentiary Table: Choice of Benzodiazepine

Study	LOE	Study Design	Methods and Outcomes	Results	Limitations
McMullan 2014[2]	II	Experimental pharmaco-stability study	8 total study boxes containing diazepam, lorazepam, and midazolam were transported in EMS units for 120 days during the summer with temps logged every minute. Drug concentration was tested at 30 day intervals.	192 samples were collected. After 120 days, mean relative concentrations of diazepam, midazolam, and lorazepam were 97%, 99%, and 86.5%. Mean kinetic temp was 31.6°C. Increasing MKT was associated with greater degradation of lorazepam, but not	No baseline samples obtained. Performed redundant single measurement, but not duplicate testing on each sample to assess reliability of test. Storage temp may not be representative of other geographic

				midazolam or diazepam.	areas.
Clemency 2014[3]	III	Retrospective chart review	440 subjects were identified over 29 months who received 577 administrations of diazepam IV/IM or midazolam IV/IM for treatment of seizures. Primary outcome measured was medication effectiveness in cessation of seizure without repeat seizure prior to ED arrival.	237 patients received 329 doses of parenteral diazepam, 64 (27%) first dose IM. 203 patients received 248 doses of parenteral midazolam, 71 (35%) first dose IM. Midazolam was more successful in seizure cessation overall (65% v 49%), and first dose IM (69% v 25%). There was no difference with IV first dose (62% v 58%).	Retrospective study, non-randomized route of administration, medication doses based on regional protocol, deviations from recommended dose occurred in 8 diazepam cases and 26 midazolam cases, no hospital follow-up performed.
Alldredge 2001[4]	I	Randomized, double-blind control trial	205 patients with prolonged/repetitive prehospital seizures (status epilepticus) were administered IV diazepam 5mg, IV lorazepam 2mg, or placebo. Primary outcome was cessation of status epilepticus (pt regained consciousness) prior to ED arrival.	66 patients received lorazepam, 68 received diazepam, 71 received placebo. Status epilepticus was terminated in more patients who received benzos (59.1% and 42.6% v 21.1%.). No difference between lorazepam and diazepam.	Definition of cessation of status epilepticus required patient regaining consciousness, medication doses set by protocol and not weight based

Bosson 2014[5]	III	Retrospective observational study	Chart review of 1584 pediatric patients presenting to the ED with seizures were identified. 214 received midazolam by EMS. Primary outcome was apnea (BVM ventilation or intubation).	71 (4.5%) patients had apnea, 44 (62%) after midazolam, 27 (38%) without midazolam. Patients were more likely to have apnea after midazolam administration if seizures stopped, or if persistently seizing without benzodiazepine given	Retrospective nature of study, not blinded, multiple routes of drug administration (IM, IV, IN), underpowered to compare rectal diazepam group
Alldredge 1995[6]	III	Retrospective observational study	38 pediatric patients with seizures lasting >15 min received either diazepam (rectal or IV) or no benzodiazepine by EMS. Outcomes were seizure duration and	45 episodes of convulsive status epilepticus were identified, 19 were treated with prehospital diazepam (9 PR, 10 IV), 24 received no medication. Prehospital diazepam use resulted in shorter seizure duration (32 min v 60 min), and fewer recurrent seizures in the ED (58% v 85%).	Not randomized or blinded, retrospective nature of study, small sample size, not powered to compare route of administration
Galustyan 2003[7]	III	Retrospective chart review	288 pediatric patients aged 0-18 yrs with chief complaint of prehospital seizure received either diazepam IV/PR 0.2-0.5mg/kg prior to January 1996, or 0.05-0.1mg/kg after the specified date	189 pediatric patients received diazepam IV/PR 0.17mg/kg (mean dose) and 99 patients received 0.13mg/kg (mean dose) Patients in higher dose group were more likely to require prehospital intubation and admission. IV diazepam group more likely than PR to require intubation. No difference in number of repeat doses or ED interventions.	Retrospective design, interventions not compared simultaneously, mean actual doses administered did were outside protocol,

Warden 2006[8]	II	Retrospective chart review	93 pediatric patients aged < 18 yrs treated by EMS for seizures received either diazepam IV 0.25mg/kg or PR 0.50mg/kg prior to 1 January 2000, or midazolam IV 0.1mg/kg or IM 0.2mg/kg after the specified date	45 pediatric patients received diazepam, 48 received midazolam. Diazepam group had higher proportion of afebrile seizure history and IV drug administration than midazolam group. No difference in rates of seizure cessation prior to ED arrival, seizure recurrence in ED, need for airway intervention, or admission rate.	Retrospective design, interventions not compared simultaneously, patient weights not consistently recorded (unable to assess accuracy of drug dosing), greater seizure history in diazepam group, higher rate IV administration in diazepam group, single pt in midazolam group received PR dose

Evidentiary Table: Route of Benzodiazepine

Study	LOE	Study Design	Methods and Outcomes	Results	Limitations
Silbergleit 2012[9]	I	Randomized, double-blind, noninferiority trial	732 patients (adults and children) with seizures lasting >5 min were randomized to IM midazolam or IV lorazepam. Primary outcome was cessation of seizures without rescue therapy upon arrival to ED.	IM midazolam (362 patients) was as effective as IV lorazepam (370 patients) in terminating seizures without rescue therapy, 329 (73.4%) vs 282 (63.4%). The medications were also similar with respect for intubation rates and recurrence of seizures within 12 hours. IM midazolam group had lower rate of hospitalization.	Large number of patients excluded due to incorrect medication dosages, pre-selected (not weight-based) medication dos

Chamberlain 1997[10]	I	Randomized, controlled trial	24 children with seizures >10 min were randomized to IM midazolam (13 patients) or IV diazepam (11 patients). Primary outcome was time to cessation of seizures.	IM midazolam group (13 patients) received medication sooner and had more rapid cessation of seizures than IV diazepam group (11 patients).	Not blinded, small sample size
Holsti 2007[11]	II	Retrospective observational study	57 pediatric patients with seizures lasting >5 min received intranasal mucosal atomized midazolam (IN-MAD) or PR diazepam. Primary outcome was presence of seizure in the ED. Secondary outcomes were total seizure time, EMS seizure duration,	IN-MAD group (39 patients) compared to PR diazepam (18 patients) had shorter prehospital seizure duration, were less likely to have a seizure in the ED, undergo ED intubation, receive seizure medications for ongoing seizures in the ED, be admitted to hospital or PICU.	Observational study/chart review, small sample size, treatment groups not studied concurrently, incomplete prehospital documentation
Welch 2015[12]	I	Randomized, double-blind, noninferiority trial	Secondary analysis of RAMPART trial for pediatric patients only. 120 pediatric patients with seizures lasting >5 min were randomized to IM midazolam or IV lorazepam. Primary outcome was seizure cessation prior to ED arrival.	Seizure cessation was achieved in 41 (68.3%) and 41 (71.6%) of patients receiving IM midazolam and IV lorazepam.	Secondary analysis, not powered to demonstrate noninferiority in pediatric subgroup, used fixed-dose protocol
Dieckmann 1994[13]	III	Retrospective chart review	324 pediatric patients aged < 18 yrs with prehospital seizures received either diazepam PR 0.2-0.5mg/kg or IV 0.1-0.3mg/kg	No difference in rates of seizure cessation with single dose or recurrence of seizures prior to ED arrival. Seizure cessation achieved with single dose in 13/16 (81%) of PR group and 100% IV group. Recurrence of seizures prior to ED arrival in 4/16 (30.8%) of PR group and 9/15 (60%) of IV	IV group was significantly older (9.1 vs 3.0 yrs), PR group had more “acute, serious underlying pathology”, administered doses did not

				group.	adhere to prespecified range
Vilke 2002[14]	III	Retrospective chart review	86 pediatric patients aged 2 mo - 14 yrs with prehospital seizures received midazolam IV 0.1mg/kg to max of 5mg, or IM 0.2mg/kg to max of 10mg	IV group had significantly greater rate of clinical improvement, therapeutic improvement reported in 47/49 (96%) IV doses and 20/25 (80%) IM doses. No difference in admission rate.	small sample size retrospective design, endpoint of intervention not well defined

Evidentiary Table: Febrile Pediatric Seizures

Study	LOE	Study Design	Methods and Outcomes	Results	Limitations
Seinfeld 2014[15]	II	Prospective observational study	199 pediatric patients with febrile seizures lasting > 30 min (febrile status epilepticus) were identified. Antiepileptic drugs (AED) administered and duration of seizures was recorded	179 patients (90%) received at least one AED, 140 (78%) required >1 AED. First AEDs administered were lorazepam in 83 (46%), diazepam in 83 (46%), midazolam in 6 (3%), fosphenytoin in 2 (1%) and phenobarbital in 1 (0.5%) of patients. Those who received respiratory support had longer median seizure duration and received more AEDs. Longer seizure duration was associated with longer time before administration of AED.	Incomplete drug dosage and/or route documented, observational study without control group
Lahat 2000[16]	I	Prospective randomized study	53 pediatric patients aged 6 mo - 5 yrs with prolonged (>10 min) febrile seizure were	21 patients received Intranasal midazolam for 26 episodes of febrile seizures, and 23 patients received IV diazepam	Unable to differentiate between spontaneous seizure

			pre-randomized to receive either intranasal midazolam 0.2 mg/kg or IV diazepam 0.3 mg/kg (max dose 10mg)	for 26 episodes. 23/26 seizures responded to initial treatment with midazolam, and 24/26 by diazepam. Time from arrival at ED to drug administration (3.5 vs 5.5 min), and overall time from arrival at ED to seizure cessation (6.1 vs 8.0 min) was faster in midazolam group. Time from drug administration to seizure cessation only (2.5 vs 3.1 min) was faster in diazepam group.	cessation and medication action, unclear bioavailability of intranasal midazolam in patients with respiratory tract infections, unmeasured variables include IV start time and medication prep time
Rainbow 2002[17]	II	Retrospective, before-and-after observational study	107 pediatric patients received either PR/IV diazepam or IM/IV midazolam by paramedics prior to hospital arrival.	62 patients (31 febrile) received diazepam (57.9%) and 45 (15 febrile) received midazolam (43.9%). No difference in seizure cessation within 5 min (37.1% diazepam and 51.1% midazolam)	drug comparisons separated in time over 4 years, 3 different routes of administration used with different bioavailabilities

Evidentiary Table: Eclampsia

Study	LOE	Study Design	Methods and Outcomes	Results	Limitations
Crowther 1990[18]	I	Prospective randomized controlled study	51 eclamptic patients were randomly assigned to receive either 4g IV Magnesium sulphate followed by 10g IM, or 10mg IV diazepam followed by an IV infusion. For recurrent seizures, an additional 2g IV MgSO4 and 5g IM every 4 hours was	24 patients received magnesium sulphate and 27 received diazepam. There was no statistical difference in seizure recurrence.	17 patients in each arm (67% overall) received emergency anticonvulsant therapy with diazepam prior to enrollment, small overall sample size

			administered, or diazepam was titrated, respectively.	Fewer infants born in the MgSO4 group had Apgar scores <7.	
Collab Trial 1995[19]	I	Prospective multicenter randomized trial	1,687 eclamptic patients were randomized to either magnesium sulphate IV/IM vs diazepam IV (910 patients) at 23 centers, or magnesium sulphate IV/IM vs phenytoin IV (777 patients) at 4 centers. MgSO4 dose was 4g IV followed by IV infusion or repeated IM injections. Diazepam dose was 10mg IV followed by IV infusion. Phenytoin was only given for seizure prevention in patients after diazepam.	MgSO4 group had fewer recurrent seizures and 52% lower risk of recurrent seizures than diazepam, 67% lower risk than phenytoin. No difference in maternal mortality between MgSO4 and diazepam or MgSO and phenytoin.	Trial conducted in developing countries, phenytoin group also received diazepam, substantial number of patients randomized received more than one anticonvulsant

1. Beskind DL, Rhodes SM, Stolz U, et al. When should you test for and treat hypoglycemia in prehospital seizure patients? Prehospital emergency care : official journal of the National Association of EMS Physicians and the National Association of State EMS Directors 2014;18:433-41.
2. McMullan JT, Jones E, Barnhart B, et al. Degradation of benzodiazepines after 120 days of EMS deployment. Prehospital emergency care : official journal of the National Association of EMS Physicians and the National Association of State EMS Directors 2014;18:368-74.
3. Clemency BM, Ott JA, Tanski CT, Bart JA, Lindstrom HA. Parenteral midazolam is superior to diazepam for treatment of prehospital seizures. Prehospital emergency care : official journal of the National Association of EMS Physicians and the National Association of State EMS Directors 2015;19:218-23.
4. Alldredge BK, Gelb AM, Isaacs SM, et al. A comparison of lorazepam, diazepam, and placebo for the treatment of out-of-hospital status epilepticus. The New England journal of medicine 2001;345:631-7.

5. Bosson N, Santillanes G, Kaji AH, et al. Risk factors for apnea in pediatric patients transported by paramedics for out-of-hospital seizure. Annals of emergency medicine 2014;63:302-8 e1.
6. Alldredge BK, Wall DB, Ferriero DM. Effect of prehospital treatment on the outcome of status epilepticus in children. Pediatric neurology 1995;12:213-6.
7. Galustyan SG, Walsh-Kelly CM, Szewczuga D, Bergholte J, Hennes H. The short-term outcome of seizure management by prehospital personnel: a comparison of two protocols. Pediatric emergency care 2003;19:221-5.
8. Warden CR, Frederick C. Midazolam and diazepam for pediatric seizures in the prehospital setting. Prehospital emergency care : official journal of the National Association of EMS Physicians and the National Association of State EMS Directors 2006;10:463-7.
9. Silbergleit R, Durkalski V, Lowenstein D, et al. Intramuscular versus intravenous therapy for prehospital status epilepticus. The New England journal of medicine 2012;366:591-600.
10. Chamberlain JM, Altieri MA, Futterman C, Young GM, Ochsenschlager DW, Waisman Y. A prospective, randomized study comparing intramuscular midazolam with intravenous diazepam for the treatment of seizures in children. Pediatric emergency care 1997;13:92-4.
11. Holsti M, Sill BL, Firth SD, Filloux FM, Joyce SM, Furnival RA. Prehospital intranasal midazolam for the treatment of pediatric seizures. Pediatric emergency care 2007;23:148-53.
12. Welch RD, Nicholas K, Durkalski-Mauldin VL, et al. Intramuscular midazolam versus intravenous lorazepam for the prehospital treatment of status epilepticus in the pediatric population. Epilepsia 2015;56:254-62.
13. Dieckmann RA. Rectal diazepam for prehospital pediatric status epilepticus. Annals of emergency medicine 1994;23:216-24.
14. Vilke GM, Sharieff GQ, Marino A, Gerhart AE, Chan TC. Midazolam for the treatment of out-of-hospital pediatric seizures. Prehospital emergency care : official journal of the National Association of EMS Physicians and the National Association of State EMS Directors 2002;6:215-7.
15. Seinfeld S, Shinnar S, Sun S, et al. Emergency management of febrile status epilepticus: results of the FEBSTAT study. Epilepsia 2014;55:388-95.
16. Lahat E, Goldman M, Barr J, Bistritzer T, Berkovitch M. Comparison of intranasal midazolam with intravenous diazepam for treating febrile seizures in children: prospective randomised study. Bmj 2000;321:83-6.
17. Rainbow J, Browne GJ, Lam LT. Controlling seizures in the prehospital setting: diazepam or midazolam? Journal of paediatrics and child health 2002;38:582-6.
18. Crowther C. Magnesium sulphate versus diazepam in the management of eclampsia: a randomized controlled trial. British journal of obstetrics and gynaecology 1990;97:110-7.
19. Which anticonvulsant for women with eclampsia? Evidence from the Collaborative Eclampsia Trial. Lancet 1995;345:1455-63.
